# Precipitation Law of Vanadium in Microalloyed Steel and Its Performance Influencing Factors

**DOI:** 10.3390/ma15228146

**Published:** 2022-11-17

**Authors:** Hongliang Liu, Bo Yang, Yu Chen, Chuncheng Li, Chengjun Liu

**Affiliations:** 1 Technology Research Institute of Bengang Steel Plates Co., Ltd., Benxi 117000, China; 2 Key Laboratory of Ecological Metallurgy of Multi-metal Symbiosis Ore, School of Metallurgy, Northeastern University, Shenyang 110004, China

**Keywords:** microalloyed steel, precipitation strength, microstructure, intensity

## Abstract

Based on theoretical calculations, laboratory simulation research and industrial production data analysis combined with characterisations such as metallographic microscope, scanning electron microscope (SEM), transmission electron microscope (TEM) and microhardness testing, this study investigated the state of occurrence and the precipitation law of vanadium (V) in microalloyed steel to determine a reasonable production process for V microalloyed steel. The results showed that the V(C,N) precipitation phase was the main form of V in microalloyed steel that precipitated after the transformation of austenite to ferrite. The amount of V precipitation was positively correlated with the amount of V that was added. However, the precipitation temperature was not significantly correlated with the amount added. When the V content increased from 0.03% to 0.06%, the initial precipitation temperature only increased by 23 °C. The coiling temperature was identified as the core factor affecting the strength of V microalloyed steel. When the effects of precipitation strengthening and microstructure strengthening were considered, as the coiling temperature decreased, the strength first increased, then decreased and finally increased again. Under different processing conditions, the strengthening of vanadium in the material increased first and then decreased as the temperature decreased (700–200 °C). The corresponding temperatures for the best strengthening effect of aging treatment, industrial statistical data and simulating coiling were 550, 470 and 400 °C, respectively. The difference between laboratory research results and industrial production was found. When V precipitation strengthening was used to improve material properties, it was necessary to determine a reasonable quantity of V to add and the production process, according to different alloy systems, to make more effective use of V microalloyed resources.

## 1. Introduction

The concept of the microalloying of steel began in the 1960s [1]. Alloying with strong carbonitride formers as Nb, V and Ti is one of the significant methods used to successfully develop new microalloyed steel products with superior properties [2,3,4]. The outstanding features of microalloyed steels, such as energy and cost saving, good mechanical properties and easy fabrication inspire extensive research and considerable development [5].

The influence of vanadium microalloying on the microstructure and properties of microalloyed steels were carried out by many researchers [6,7,8,9,10,11,12,13,14]. In the work of Hu and Zhu [6], Martensitic stainless steel with different vanadium contents (0–1.0 wt.%) was investigated, and they found that the microstructures with different V content were different. Ollilainen and Kasprzak et al. [7] found that steel containing higher levels of vanadium had a higher volume fraction of proeutectoid ferrite. The microhardness of proeutectoid ferrite was between 213 and 334 HV0.2 and was increased with silicon and vanadium additions. Fang et al. studied the precipitation temperatures and stability of V(C,N) and Ti(C,N) [8]. The precipitation kinetics of vanadium carbides and its interaction with the austenite-to-ferrite phase transformation was studied in the work of Ioannidou et al. [9]. The randomly dispersed precipitation and interphase precipitation (Ti, Mo, V)C particles coexisted in the Ti-Mo-V steel. When the temperature was higher than 872 °C, the addition of vanadium could increase the driving force for (Ti, Mo, V)C precipitation in austenite, resulting in an increased nucleation rate and shortened incubation period, promoting the (Ti, Mo, V)C precipitation [10]. The results of Ren et al. [11] showed that there was a minor increase in the yield strength by the vanadium addition of 0.31 wt.%, but the high vanadium addition of 0.6 wt.% could greatly enhance the yield strength by 113 MPa. The grain refinement and solid solution strengthening effect of vanadium were rather weak. Li et al. [12] found that the yield strength increased with increase in V content. Especially, the steel with 0.1 wt.% V had the most efficient strengthening effect, and its yield strength increased nearly 370 MPa compared with that of steel without V. Pelligra et al. [13] found that the homogenisation of a microstrain between ferrite and martensite was locally enhanced and the strain gradients at the ferrite/martensite (F/M) interfaces reduced in the V-added DP1300 steel. Ultrafine austenite grains with average size of 2 um were obtained by combining thermomechanical control process followed by reheating in a vanadium microalloyed steel in the work of Yang et al. [14].

The dissolution and precipitation laws of V in microalloyed steel are the core technology that affects its microalloying effect. At present, most of the research reports on V microalloying are based on laboratory research results, and the lack of industrial data support and verification. This study focused on the precipitation behaviour of V combined with industrial production data for statistical analysis, in order to investigate the industrial production scheme for the effective utilisation of V resources. A comprehensive analysis of the precipitation characteristics and the microstructure properties of V microalloys will be significant in guiding the effective utilisation of V resources, as well as the industrial production adjustment of alloy composition and process parameters, in order to provide a theoretical basis for the industrial production of V microalloyed steel.

## 2. Materials and Methods

### 2.1. Sample Preparation

In order to compare and analyse the effect of V microalloys, two sets of samples were prepared. Sample one was an industrial production with 0.05 wt.% V content and sample two was a control sample that was smelted without any V added. The specific composition is shown in Table 1. 

Sample one was pretreated with molten iron, smelted in a converter and then subjected to Ladle Furnace (LF) and Rheinstahl–Heraeus (RH) refining treatments to remove residual elements in the steel. The percentage of nitrogen of sample one was less than 60ppm. Subsequently, the sample was made into a slab by continuous casting. The slab was heated to 1200 °C in a furnace. This was followed by controlled rolling via the thermal mechanical controlled processing (TMCP) hot rolling process to obtain the final product for further research. The thickness of the slab and the final rolled product were 210 mm and 14.7 mm, respectively.

Sample two was smelted in an intermediate frequency induction furnace with commercial pure iron as the smelting masterbatch. Ferrosilicon, ferromanganese, ferroniobium, ferrotitanium, graphite, etc., were appropriately added, followed by deoxidation with aluminium. In theory, the content of sample two was higher than sample one, because it had not been refined. From the ingot process, a steel billet (75 mm × 75 mm) was forged using an air hammer (750 kg), then rolled into a steel plate (12 mm thick) using a small rolling mill, for the comparative study of heat treatment.

Sample one was processed to a cylinder with a diameter of 8 mm and a length of 15 mm to be used in the coiling temperature simulation research. Sample one and sample two were cut to a size of 10 mm × 10 mm × 15 mm for the heat treatment experiment, characterisation and analysis of the V precipitation temperature. The industrial statistical results of this study were collected from the industrial production data of sample one.

### 2.2. Experimental Procedure

**Figure 1:**
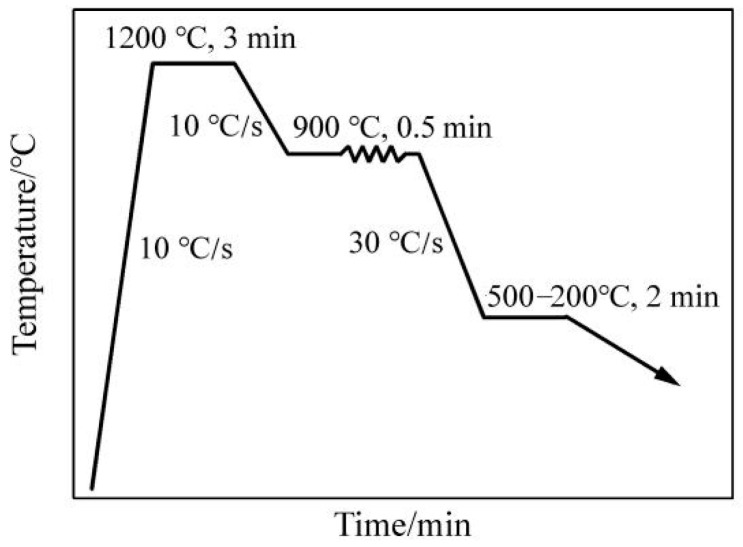
Schematic diagram of thermal simulation process.

The influence of different coiling temperatures on the microstructure of V microalloyed steel was investigated using a Gleeble-2000 thermal simulator. First, the steel was heated to 1200 °C, at a heating rate of 10 °C/s, held at that temperature for 3 min and then cooled to 900 °C, at a cooling rate of 10 °C/s, and held for 30 s, where the strain was 0.5 and the deformation rate was 1s^−1^. Second, the sample was cooled to 500 °C, 450 °C, 400 °C, 300 °C and 200 °C in turn and held at each of these temperatures for two minutes. It was then further cooled to 200 °C at a cooling rate of 2 °C/s. The specific simulation process is shown in Figure 1.

**Figure 2:**
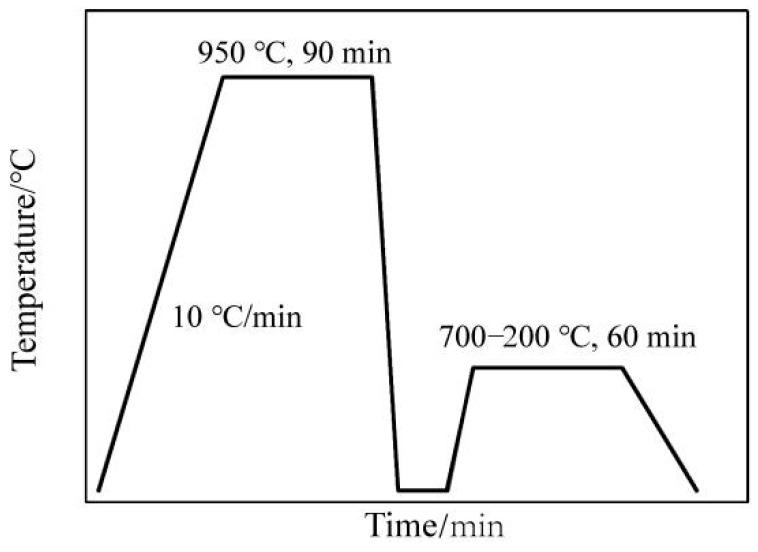
Thermal solving and aging process of vanadium.

In order to study the precipitation laws of microalloyed V in steel, the samples with and without the addition of V were pretreated with an aging treatment by a muffle furnace. V microalloys need to be fully dissolved to eliminate the original microstructure characteristics of the two materials. After this, the V precipitation was promoted by tempering the sample. The difference in hardness between the two materials was measured by the microhardness test to evaluate the V precipitation behaviour. The specific process is as follows: the samples were heated to 950 °C and then quenched, after holding that temperature for 1.5 h. They were then heated to 200–750 °C and held there for 1 h, and were finally air cooled to room temperature. The thermal solving and aging process of vanadium is shown in Figure 2.

### 2.3. Characterization methods

The microstructure of the prepared materials was analysed by a metallographic microscope (OLYMPUS-BX51, Tokyo, Japan) and a scanning electron microscope (SEM, SSX-550, Zeiss, Oberkochen, Germany). A thermodynamic analysis was conducted by using FactSage 8.0 software. The microhardness test was performed on an HVS-1000 microhardness tester (Times Peak Technology Co., Ltd., Beijing, China) to measure nine points of microhardness data in any two vertical directions. The microstructure analysis was processed by a transmission electron microscope (TEM, TECNAI-G20, Fei, Hillsboro, OR, USA) to analyse the precipitation phase, where the sample was wire cut into 300 μm flakes, ground into 100 μm flakes and finally treated by a double-spray method.

The mechanical test of the material in industrial production was performed on a CMT30 microcomputer control electron universal testing machine (ASTM, Philadelphia, PA, USA). The coiling samples were collected along the rolling direction after air cooling for eight hours. The test temperature was 25 °C and the results were obtained through the test software (DSC-10) in accordance with GB/T228.1-2010 (metallic material tensile test method at room temperature). 

## 3. Results

### 3.1. Occurrence State of V in Microalloyed Steel

**Figure 3:**
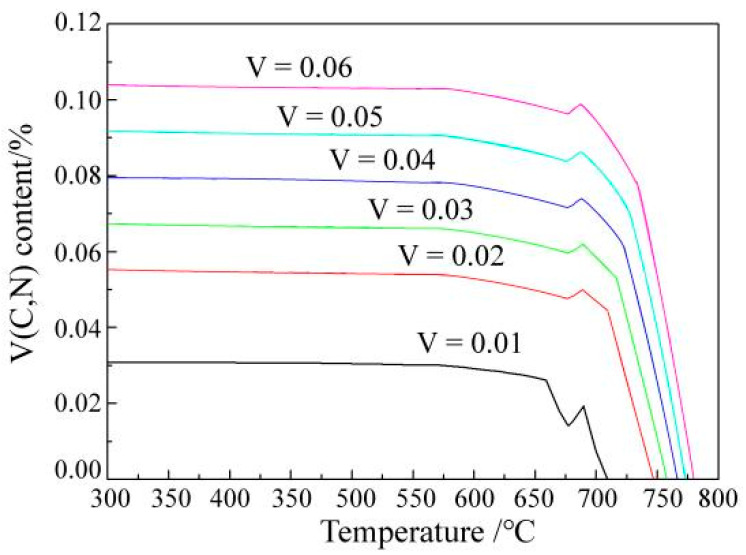
Calculated dissolution and precipitation laws of the different V contents in the samples.

To study the occurrence state of V in alloy, FactSage thermodynamics software was used to analyse the dissolution and precipitation laws of V in steel containing different quantities of V. The results are shown in Figure 3.

The results of the calculations showed that the precipitation amount of V was proportional to the V contained in the sample and the precipitation temperature increased with the increase in the V content. However, even if the V content increased to 0.06 wt.%, the precipitation temperature was still below 800 °C. When the V content increased from 0.03–0.06 wt.%, the precipitation temperature increased from 757–780 °C.

According to the phase transition analysis, the precipitation of V mainly occurred during the transformation from austenite to ferrite and the V content had some influence on the maximum temperature of V precipitation. All of the V had completely precipitated at 600 °C, regardless of the V content. Even if the temperature decreased, the precipitation quantity of V would no longer increase. Thermodynamic analysis showed that the dissolution and precipitation temperature of V in steel was stable and the effect of V microalloyed steel on material performance was mainly related to V content, which was consistent with previous research [15,16]. Furthermore, the performance statistics of V microalloyed steels with different compositions in industrial production were also consistent with the theoretical analysis results. These illustrated that the strength of the material, especially the yield strength, was significantly improved with the increase in the V content.

### 3.2. Test Result Regarding the Occurrence State of V

The thermodynamic data showed that the temperature at which a large amount of V precipitated was after the ferrite transformation process. Theoretically, as the temperature decreases, the thermodynamic conditions become beneficial for V precipitation. However, the diffusion of alloys, such as C and V, was also affected by the decrease in temperature [15]. Therefore, from the perspective of kinetics, a low temperature was unfavourable for V precipitation. Combining thermodynamic and kinetic factors, the theoretically optimal temperature range for V precipitation in ferrite should be lower than 600 °C.

**Figure 4:**
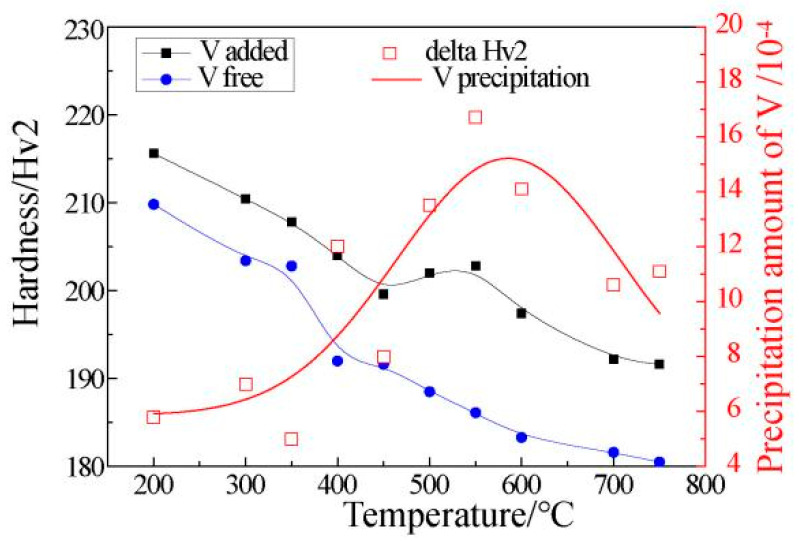
Relationship between precipitation amount of V and tempering temperature.

To systematically investigate the precipitation laws of V, this research referred to the method of Misra [17], adopting quenching and tempering approaches as well as microhardness testing. First, the sample was heated to above 950 °C to transform its microstructure into austenite. It was then held at that temperature for 1.5 h to ensure that all of the V was in solid form. This state was then maintained by a further quenching treatment. V was then reprecipitated after tempering at different temperatures. The precipitation formed in the tempered martensite will directly affect the microhardness of the structure. By comparing the hardness of microalloyed samples with and without the addition of V, after aging treatment at different temperatures, the amount of V precipitation can be determined. The result is shown in Figure 4, and the data fitting conforms to the characteristic of a parabola. The amount of V precipitation reached its maximum value only within a suitable temperature range. Although the kinetic conditions were better at high temperatures, the thermodynamic conditions were insufficient, which resulted in an incomplete precipitation of V and low material hardness. At low temperatures, the precipitation was also incomplete. This was owing to insufficient kinetic conditions. Thus, the optimal precipitation temperature of V was 550 °C.

In order to fully utilise the effect of V precipitation strengthening when using V microalloys to improve material strength, the relationship between the precipitation temperature and precipitation amount of V should be fully considered when formulating the coiling temperature process.

### 3.3. The Results of Thermal Simulation Testing

**Figure 5:**
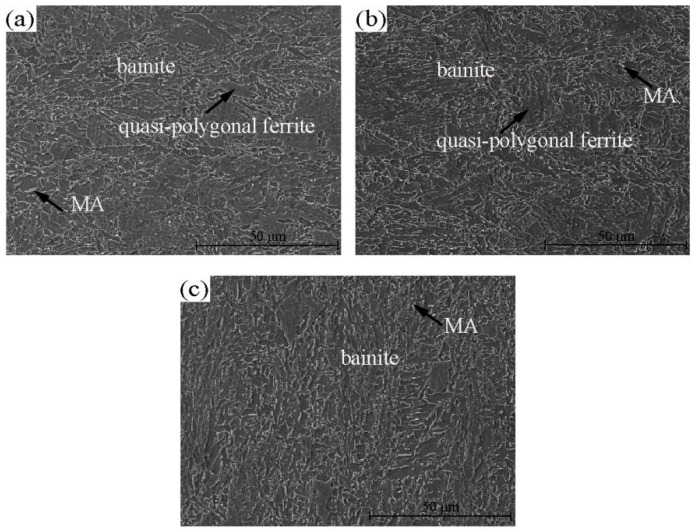
Microstructures of the samples at different coiling temperatures: (**a**) 500 °C, (**b**) 400 °C and (**c**) 300 °C.

The occurrence state of V microalloy in steel is the core technique for the effective utilisation of V microalloyed steel. The analysis of the results illustrated that V precipitated in the ferrite microstructure. Therefore, the coiling temperature was quite significant for the precipitation of V in industrial production. However, the microstructure of microalloyed steel will change with the adjustment of the coiling temperature. Therefore, controlling the microstructure characteristics and strengthening precipitation were an important basis for the formulation of the production process of microalloyed steel. In this study, a Gleeble-2000 thermal simulation testing machine was used to simulate different coiling temperatures. The process is shown in Figure 1. Typical structures of simulated samples were characterised by SEM analysis as shown in Figure 5. The Image-Pro Plus software was used to measure percentage of phases in different heat treatment temperatures.

The results showed that granular bainite was the main component of the coiling sample at 500 °C. In addition, large quasipolygonal ferrite, a fine MA component and a large microstructure can also be observed. When the temperature dropped to 400 °C, the structure was still mainly granular bainite, while the amount of quasipolygonal ferrite decreased significantly and bainite and ferrite appeared in some regions. In addition, the content of the MA component increased, and the grain size decreased. When the temperature was 300 °C, the characteristics of bainite and ferrite were noticeable, and the quantity of the MA component increased. Therefore, with the decrease in the coiling temperature, due to the inhibition of precipitation relative to austenite grain growth, the austenite grain size becomes significantly smaller, which is consistent with the research results of Khalaj et al. [18]. The amount and size of quasipolygonal ferrite decreased, while the content of the MA component increased significantly. The material strength gradually increased with the decrease in the coiling temperature [19,20]. 

**Figure 6:**
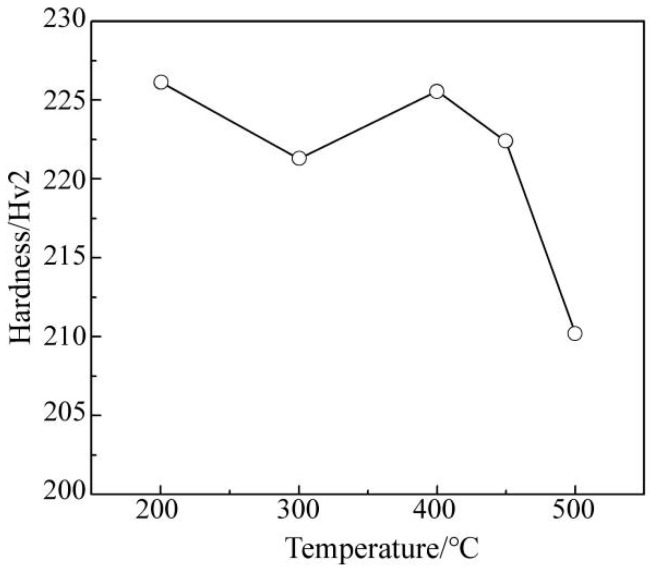
Hardness of the samples at different coiling temperatures.

The microhardness test was carried out for the samples taken at different simulated temperatures and the data are shown in Figure 6. The results showed that the difference in microhardness was nonlinear and that the maximum hardness was obtained at 400 °C and 200 °C, which was inconsistent with the results of microstructure analysis.

The material hardness is determined by macrostructure characteristics and the microprecipitate phase. The macrostructure analysis had obvious differences under different coiling temperatures, which is the main factor affecting the strength of the material. When the temperature was 200 °C, the proportion of martensite in the structure increased, leading to the increase in material hardness [21]. When the temperature was 400 °C, it was conducive to the precipitation of V in the structure, so the material hardness was close to the coiling temperature of 200 °C. However, the previous analyses showed that the coiling temperature also had a significant effect on the V precipitation behaviour, which also affected the material strength. Therefore, the mechanical properties of V microalloyed steel were determined by both macrostructure and precipitation behaviour [22].

### 3.4. Industrial Production Data

**Figure 7:**
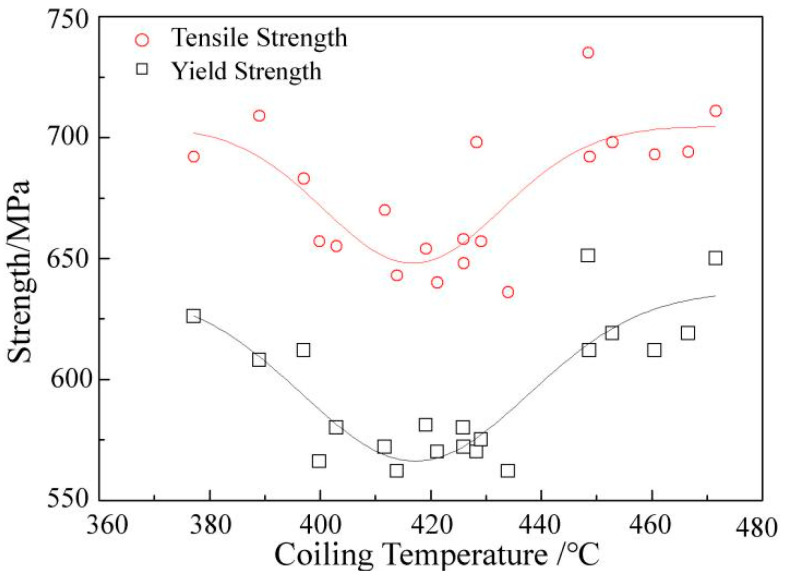
The relationship between the material strength and the coiling temperature.

To verify the effect of V microalloys in the industrial production process, the mass production data of sample one were collected and analysed. The coiling temperature was adjusted several times, owing to the abnormal changes in material strength during on-site production. Through the collection of much test data, the relationship between the material strength and the coiling temperature was statistically analysed and the result is shown in Figure 7. The artificial neural network model developed by Khalaj et al. [23]. was used to predict the tensile strengths of different coiling temperature samples, and the predicted values are basically consistent with the measured values.

It was found that the coiling temperature had a significant effect on the strength of V microalloyed steel. The yield strength and the tensile strength of the material were lowest when the coiling temperature was in the range of 400–440 °C. Above or below this range, the material properties were improved. The result was consistent with the result of the microhardness test, but the extreme points were different. The coiling temperature interval of the simulated coiling process was 100°C. The minimum and maximum values were 300 °C and 400 °C, respectively. A lower range was observed in industrial production owing to massive quantities of data. In this case, the minimum and maximum values were 420 °C and 470 °C, respectively.

**Figure 8:**
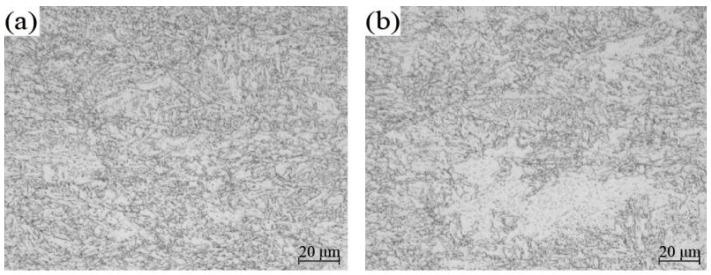
Microstructure of the samples at different coiling temperatures in industry: (**a**) 403 °C and (**b**) 467 °C.

In order to further analyse the influence of the coiling temperature on the strength, typical coiling temperature samples were selected for metallographic analysis, as shown in Figure 8. When the coiling temperature was 467 °C, the yield strength and tensile strength reached 619 MPa and 694 MPa, respectively. Furthermore, it can be observed that the metallographic structure was typical granular bainite and quasipolygonal ferrite, and some MA components. When the coiling temperature was reduced to 403 °C, the yield strength and tensile strength were 580 MPa and 650 MPa, respectively. At this time, the structure was mainly granular bainite and bainitic ferrite, accompanied by a very small amount of quasipolygonal ferrite. The strength of the coiling microstructure at 403 °C was higher than at 467 °C, owing to less quasipolygonal ferrite, more bainitic ferrite and a smaller grain size. This is consistent with the results of the simulation tests.

**Figure 9:**
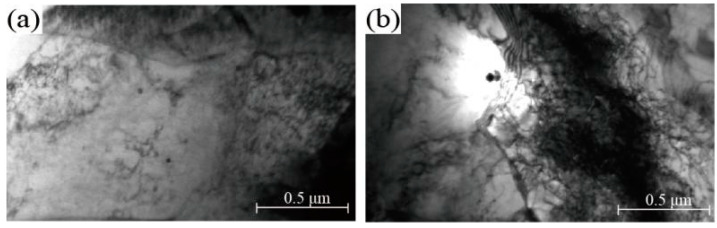
TEM images of the samples at different coiling temperatures: (**a**) 467 °C and (**b**) 403 °C.

The nonlinear change in material properties as a result of the change in the coiling temperature is related to the precipitation strengthening of V. Based on this, the coiling microstructure of the industrial production at 467 °C was analysed by TEM analysis. As shown in Figure 9a, in the ferrite lath a large amount of fine precipitation [24], mainly V(C,N), was found according to size and morphology. Figure 9b reveals the microstructure of the coiling sample at 403 °C. It can be seen that the precipitation, mainly Ti and Nb, was large in size and small in amount, illustrating that the uniformly dispersed precipitation contributed more significantly to improving the strength of the material.

## 4. Analyses and Discussions

### 4.1. Effects of V Occurrence State on Properties

As shown in Figure 4, according to the aging treatment results of microalloyed samples with and without V, it was found that the hardness of the V microalloyed sample was higher than that of its counterpart at different temperatures. For the sample without V, the hardness decreased monotonically as the tempering temperature increased. For the sample with V, the hardness decreased in the temperature ranges of 200 to 450 °C and 550 to 750 °C, and the hardness increased in the temperature range of 450 to 550 °C.

Furthermore, it can be found that V had a strengthening effect, whether in the form of a solid solution or precipitation. However, the precipitation strengthening of V was more significant. Therefore, fully utilising the precipitation strengthening of V is the core technology for the effective application of V microalloys.

There were distinct differences in the effect of V precipitation strengthening under different process conditions; however, this was related to the strength of the matrix microstructure. The content and size of the precipitation phase were also significantly different as a result of different production processes that made different contributions to material strength.

The simulation test was a coiling heat preservation process of two minutes. The macrostructure of the simulated sample was compared to that of the industrial production. It was found that the two materials had a similar macrostructure, while the grain size of the simulated sample was smaller, as shown in Figure 5 and Figure 8. Therefore, the hardness of the simulated coiling sample was relatively high, as shown in Figure 6.

Industrial products were stored in the form of coils, with a long heat preservation time and a slow cooling rate, which still remained at approximately 100 °C after eight hours. As a result, its structure was slightly coarser than that of the simulated sample but the macrostructure types in the coiling temperature range were similar. At a coiling temperature of 467 °C, the macrostructure contained more quasipolygonal ferrite that was large in size, which was not conducive to improving the material strength. However, there was more V precipitation that was small in size and dispersed in distribution, which was beneficial to improving the material strength, especially the yield strength. When the coiling temperature was 403 °C, the macrostructure of the sample became smaller, but the precipitation of V was insufficient. Because the presence of V in a solid form had less effect on the precipitation strengthening, the material strength was slightly lower. Comparing the yield ratio of coiling samples at 467 °C and 403 °C, it was also found that V precipitation strengthening can significantly improve the yield strength of the material.

### 4.2. Production Process and V Precipitation Law

Through aging treatment, simulating coiling at different temperatures and comparing the results of industrial statistical data, it was found that the change in strength (hardness) was consistent under differing process conditions as the temperature decreased. More specifically, the maximum value appeared at first, and then the minimum value appeared. However, the corresponding temperatures for extreme values of different processes were not the same, as shown in Figure 4, Figure 6 and Figure 7.

The maximum hardness value of the aging treatment, the industrial production and the simulated coiling process appeared at 550 °C, 470 °C and 400 °C, respectively. However, it was more accurate to compare the temperature of the minimum hardness value for which the corresponding minimum temperatures were 450 °C, 420 °C and 300 °C, respectively.

**Figure 10:**
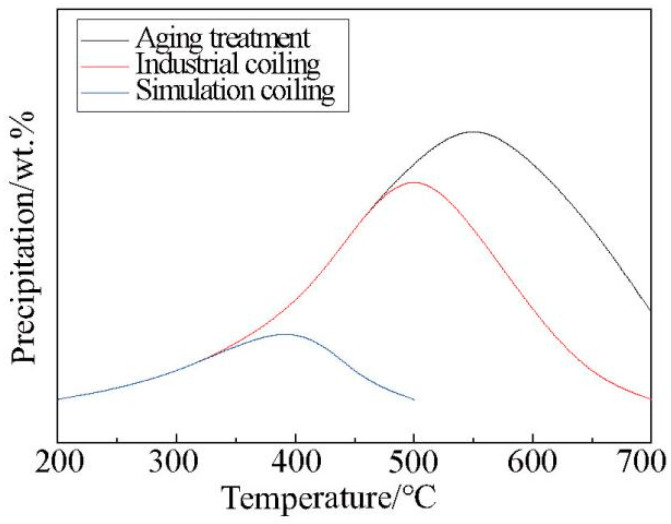
The relationship between the precipitation and process.

Considering the characteristics of the process and the microstructure comprehensively, this phenomenon can be attributed to many factors. The precipitation of V in the three processes had the maximum value under different process conditions, which conformed to the characteristics of a parabola. As shown in Figure 10, the aging treatment adopted a quenching process to ensure that V existed mostly in a solid state and then was fully precipitated by tempering. This produced the best thermodynamic conditions and the maximum precipitation amount when compared with the other processes. The strain was the largest under the industrial production process, where the slab was rolled from 210 mm–14.7 mm, which was beneficial for inducing the precipitation of V. In addition, the slow temperature drop in the coil after coiling was also conducive to V precipitation. Therefore, the industrial production process had the optimal kinetic conditions. However, there is no guarantee that V will not precipitate before coiling, because part of V will precipitate in advance due to excessive strain, thereby reducing the V content in the solid state. This means that the thermodynamic conditions of the industrial production process were insufficient, with a lower maximum temperature value when compared with aging treatment. The strain and the coiling were simulated at 200–500 °C and 900 °C, respectively, during the simulated coiling process and then held for two minutes after coiling. Therefore, both kinetics and thermodynamics were inadequate during this process. This caused the maximum value of precipitation and the lowest temperature.

The temperature change trend of the maximum hardness value was similar to that of the minimum hardness value under different processes. The industrial production process had the smallest size of precipitates owing to the sufficient kinetic conditions. The effect of this on material strength was also the most significant. Unfortunately, the research regarding the coiling temperature above 470 °C was not carried out in this study, but the fitting data showed that when the V content was 0.05 wt.%, the difference in the tensile strength of the samples in different coiling processes was greater than 57 MPa. This indicated that the production process had a significant influence on the strengthening effect of V microalloys.

### 4.3. Reasonable V Content and the Production Process

**Figure 11:**
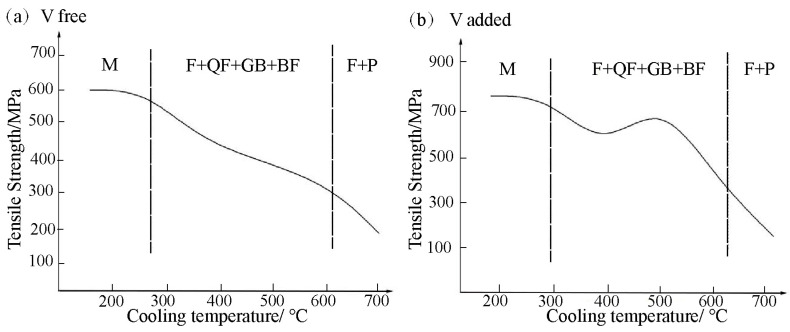
The relationship between tensile strength and coiling temperature.

Considering the effect of precipitation strengthening and microstructure strengthening in V microalloyed steel, the variation in the strength of V microalloyed steels and hot-rolled coiling temperature should be different from that without the addition of V, as shown in Figure 11. When V was not added to the microalloy, ferrite, pearlite, bainite and martensite microstructures could be obtained by decreasing the coiling temperature. The grain size also decreased, and therefore the strength of the material increased monotonously.

However, due to the precipitation strengthening of V in microalloyed steel, the refining effect of the ferrite and the pearlite microstructure was dominated, as the coiling temperature decreased in the initial stage, thereby improving the material strength. When the coiling temperature dropped below 600 °C, as a result of V precipitation and microstructure strengthening, a maximum strength value in the mixed microstructure of bainite and ferrite could be observed as the temperature decreased. With a further decrease in temperature, the precipitation of V and the precipitation strengthening was suppressed. Nevertheless, a linear increasing trend in material strength reappeared, and seemed to stable. Therefore, the strength of V microalloyed steel had both a maximum and a minimum value as the coiling temperature decreased, as shown in Figure 11b.

Based on industrial production data, the relationship between the strength and coiling temperature of V microalloyed steels with different compositions and different production processes after hot rolling was clarified. This corresponded to the different ranges shown in Figure 9b. For example, in this study, the coiling temperature in industrial production corresponded to the minimum value.

Therefore, in order to make effective use of V microalloyed resources and fully utilise the precipitation strengthening of V, it is necessary to determine the amount of V to add and the production process according to different alloy systems, and to consider the influence of coiling temperature on maximum material strength, which is a theoretical basis for the effective utilisation of V microalloys.

## 5. Conclusions

Using the metallographic microscope, scanning electron microscope (SEM), transmission electron microscope (TEM) and microhardness testing, and combining with the theoretical calculation laboratory simulation research and industrial production data analysis, the precipitation law and the effect of vanadium on the microstructures and performances in the steel were investigated in this work. The main conclusions are drawn as follows:

(1) V precipitation mainly occurred after ferrite transformation. As the V content increased, the precipitation temperature also increased. When the V content increased from 0.03 to 0.06 wt.%, the initial precipitation temperature only increased by 23 °C. Combining thermodynamics and kinetic conditions, there was an optimal temperature range for V precipitation in ferrite, and the precipitation amount was in line with the characteristics of a parabola. When the content of vanadium is 0.05 wt.%, the maximum value of V precipitation was 550 °C in this study.
(2) Under different processing conditions, the strengthening of vanadium in the material increases first and then decreases as the temperature decreased (700–200 °C). The corresponding temperatures for the best strengthening effect of aging treatment, industrial statistical data and simulating coiling were 550, 470 and 400 °C, respectively. The difference between laboratory research results and industrial production was found.
(3) An effective strength increment can be attained by the double strengthened modes of precipitation strengthening and hardened structure strengthening of vanadium in this work. The effects of precipitation and microstructure strengthening were that as the coiling temperature decreased, the material strength first increased, then decreased and finally increased again. This means that the strength of V microalloyed steel has a maximum value.
(4) Fine V(C,N), Nb(C,N) and/or Ti(C,N) were observed in the industrial steels with different coiling temperatures in the present work.

We hope that our work can provide some help for the effective utilization of vanadium in the industrial production of steel.

## Figures and Tables

**Table 1:** Chemical composition of the testing steel (wt.%).

No.	C	Si	Mn	Cr	Nb	Ti	V
1	0.075	0.20	1.65	0.25	0.040	0.025	0.052
2	0.081	0.22	1.70	0.23	0.035	0.025	--

## Data Availability

The data presented in this study are available on request from the corresponding author.
